# Interactions between atomic-scale skyrmions in 2D chiral magnets

**DOI:** 10.1038/s41598-026-41762-2

**Published:** 2026-03-10

**Authors:** Mai Kameda, Koji Kobayashi, Yuki Kawaguchi

**Affiliations:** 1 Toyota Central R&D Labs., Inc., Nagakute, 480-1192 Japan; 2 Department of Physics, Sophia University, Chiyoda-ku, Tokyo 102-8554 Japan; 3 Department of Applied Physics, Nagoya University, Nagoya, 464-8603 Japan; 4 Research Center for Crystalline Materials Engineering, Nagoya University, Nagoya, 464-8603 Japan

**Keywords:** Materials science, Physics

## Abstract

Skyrmions are topologically stable spin textures that hold great promise as information carriers in next-generation magnetic memories. Recently, skyrmions only a few nanometers in radius have been observed in several materials, opening a path toward ultrahigh-density integration. As a step toward improving their controllability, we numerically investigate interactions between atomic-scale skyrmions embedded in a uniformly magnetized background of two-dimensional chiral magnets, under tilted magnetic fields and magneto-crystalline anisotropy. We find that attractive potential wells, predicted for larger skyrmions from shape deformation, persist even at the atomic scale. As skyrmions shrink, the short-range repulsion is enhanced, while a tilted background magnetization increases the attraction at larger separations. Under strong magneto-crystalline anisotropy, a magnetic domain forms between skyrmions, producing a deep attractive well whose position and depth are nearly independent of skyrmion size. This shows that tightly bound skyrmion pairs with exchange-scale energies can exist even at the atomic scale. Furthermore, under the magneto-crystalline anisotropy, the atomic lattice potential increasingly affects smaller skyrmions, pinning them and suppressing motion despite attraction. These findings deepen understanding of inter-skyrmion interactions across scales and lay the groundwork for controlling atomic-scale skyrmions in future device technologies.

## Introduction

Skyrmions, topologically stable magnetic vortices, are potentially capable of carrying information in magnetic memories and computing devices^1–12^. Representative examples include skyrmion racetrack memory^13,14^ and neuromorphic computing devices^15–17^, many of which rely on current-driven skyrmion motion to transport information and control synaptic weights. A key factor governing their controllability is the manipulation of inter-skyrmion interactions, which directly affect skyrmion motion. In this context, extensive research has been conducted on both skyrmion-skyrmion interactions and their role in current-driven skyrmion dynamics. Conventional circular skyrmions have only inter-skyrmion repulsion^6,18^. For current-driven motion of circular skyrmions in nanotracks, they repel each other while conserving their number and also experience repulsion from edges^19,20^. This repulsion helps skyrmions avoid defects in nanotracks^21,22^, but can also cause clogging of skyrmion bits, indicating the need to maintain proper spacing for stable memory operation^23^.

Recently, inter-skyrmion attraction is found to appear due to various factors^24–30^: frustrated exchange interactions in ultra-thin magnetic films, the background conical phase of non-centrosymmetric magnets, tilted ferromagnetic phases of polar magnets with easy-plane anisotropy, and the background helical phase of chiral magnets. The formation of skyrmion clusters under a conical background or a uniformly tilted in-plane magnetic field has also been reported^31–33^. Current-driven motion under such attractive inter-skyrmion potential might offer greater manipulability. Studies on such systems remain limited, however: the skyrmion clusters can be driven by electric currents and show a finite skyrmion Hall angle^32,33^, similar to repulsive skyrmions.

For high-density integration of skyrmion magnetic memories, one may utilize recently found small-sized skyrmions, with their radii less than $\sim 3$ nm^34–41^. Understanding how such atomic-scale skyrmions interact is crucial for controlling their motion. However, most previous studies on skyrmion-skyrmion interactions have focused on those with sizes of hundreds of nanometers or on analyses based on continuum models^26–30^. While a few works have employed lattice models, these have been limited to skyrmions with relatively large radii^24,25,30^. At present, the interaction potential of small skyrmions with radii of five sites or fewer remains unclear.

In this work, we investigate the interaction potentials between atomic-scale skyrmions embedded in uniformly magnetized two-dimensional chiral magnets. Specifically, the term “atomic-scale skyrmions” refers to skyrmions whose characteristic size is comparable to the lattice constant in a minimal lattice model, rather than those stabilized by frustrated or higher-order exchange interactions. In our previous study^30^, we have found an inter-skyrmion attractive potential appearing at skyrmion-scale separations, due to skyrmion deformation and magnetic-domain formation. The potentials exhibit hard-core repulsion at short distances and an attractive well at intermediate distances. In particular, the attractive well reaches a depth on the order of the exchange interaction when it arises from magnetic-domain formation. Here, we demonstrate that both mechanisms underlying the attractive well remain valid even for atomic-scale skyrmions, suggesting controlling skyrmions via attractive interactions could be feasible even in higher-density devices. When the attractive well arises from skyrmion deformation, the potential at large separations is well reproduced by the approximate one derived from a single-skyrmion configuration. At long distances scaled by the skyrmion size, a deeper attraction appears under a tilted magnetization. At short distances scaled by skyrmion size, smaller skyrmions exhibit stronger repulsion. In contrast, when the attractive well arises from magnetic-domain formation, the interaction potential exhibits a nearly universal form: it is almost independent of the skyrmion radius when expressed as a function of the normalized skyrmion separation. This demonstrates that strongly bound skyrmion pairs, with a binding energy on the order of the exchange interaction, can emerge even at the atomic scale. These results highlight the robustness of the attractive mechanisms across different skyrmion sizes. Our findings provide guidance for the design of nanoscale spintronic devices, such as the high-density integration of skyrmion-based magnetic memories and neuromorphic computing devices, where inter-skyrmion interactions play a crucial role.

A further characteristic feature that emerges for atomic-scale skyrmions is the influence of the underlying atomic lattice on their dynamics^42^. For small skyrmions, the magnetization directions at neighboring lattice sites differ significantly, making the energy sensitive to their relative positioning. Our simulations show that the energy depends on whether the skyrmion center coincides with a lattice site. In the presence of magneto-crystalline anisotropy, this energy difference increases as the skyrmion size decreases. Under such conditions, even strong attractions cannot overcome the lattice pinning, preventing the skyrmions from reducing their separation. Specifically, while relatively large skyrmions approach each other when placed beyond the optimal distance, smaller skyrmions keep their original spacing under the same conditions.

## Results

### Model

 We consider a two-dimensional chiral magnet, described by the classical spin Hamiltonian

1 $$\begin{aligned} H& = -J\sum _{{\boldsymbol{r}}} {\boldsymbol{S}}_{{\boldsymbol{r}}}\cdot ({\boldsymbol{S}}_{{\boldsymbol{r}}+ {\boldsymbol{e}}_x}+{\boldsymbol{S}}_{{\boldsymbol{r}}+ {\boldsymbol{e}}_y})\\ & \quad -D\sum _{{\boldsymbol{r}}} ({\boldsymbol{S}}_{{\boldsymbol{r}}}\times {\boldsymbol{S}}_{{\boldsymbol{r}}+ {\boldsymbol{e}}_x} \cdot {\boldsymbol{e}}_x +{\boldsymbol{S}}_{{\boldsymbol{r}}}\times {\boldsymbol{S}}_{{\boldsymbol{r}}+ {\boldsymbol{e}}_y} \cdot {\boldsymbol{e}}_y)\\ & \quad -B\sum _{{\boldsymbol{r}}}( S_{\boldsymbol{r}}^z\cos \phi +S_{\boldsymbol{r}}^x\sin \phi ) \\ &\quad +A\sum _{{\boldsymbol{r}}} \left[ (S^x_{\boldsymbol{r}})^4+\frac{(S^y_{\boldsymbol{r}}+S^z_{\boldsymbol{r}})^4}{4}+\frac{(-S^y_{\boldsymbol{r}}+S^z_{\boldsymbol{r}})^4}{4}\right] , \end{aligned}$$

where the magnetization ${\boldsymbol{S}}_{{\boldsymbol{r}}}$ is on a square lattice ${\boldsymbol{r}}\in \{an_x{\boldsymbol{e}}_x+an_y{\boldsymbol{e}}_y\,|\,n_x,n_y\in \mathbb {Z}\}$, *J*, *D*, *B*, and $A>0$ are the coefficients of the exchange interaction, Dzyaloshinskii–Moriya (DM) interaction^43,44^, Zeeman interaction, and the magneto-crystalline anisotropy, respectively, and $\phi$ represents the tilt angle of the external magnetic field with respect to the normal to the two-dimensional plane, the *z*-axis. The first three terms represent the minimal interactions required for the skyrmion phase. When the magnetic field is smaller than the first threshold $B < B_\textrm{cr1}$, the ground state is helical with a wavenumber determined by the ratio *D*/*J*. When the field exceeds $B_\textrm{cr1}$, a skyrmion lattice appears. Upon further increasing the magnetic field beyond the second threshold $B > B_\textrm{cr2}$, the system becomes uniformly magnetized along the field direction, and skyrmions exist as excitations embedded in this uniformly magnetized background.

We additionally consider the cubic magneto-crystalline anisotropy^31,45–51^ in a two-dimensional film cut from a cubic crystal along the (011) plane. Assuming that the magnetic hard axis lies along the original crystal axes, the magnetic anisotropy can be rewritten as shown in the last term of Eq. (1). Although the lattice on the (011) plane is not square, we use a square lattice for simplicity, while incorporating the magnetic anisotropy specific to the (011) plane. This magneto-crystalline anisotropy affects the orientation of the background magnetization^30^ at $B>B_\textrm{cr2}$. When $A=0$, the background magnetization points along the applied magnetic field due to the Zeeman interaction. In contrast, the magneto-crystalline anisotropy alone favors magnetization lying either in the *x*-*z* or *x*-*y* plane for $A>0$. As a consequence of the competition between these two energies, when the anisotropy strength exceeds a critical value and $\phi$ is small, the magnetization tilts from the field direction toward the *x* axis. In particular, for $\phi = 0^\circ$, the uniform and stable magnetization structure deviates from the $+z$ direction (the field direction) when $A/B > 0.5$, and the magnetization points along one of two degenerate directions, $\boldsymbol{t}_+$ and $\boldsymbol{t}_-$, tilted towards the $+x$ or $-x$ direction. This work focuses on examining how interaction mechanisms established for large skyrmions carry over to the small-skyrmion regime. Accordingly, Eq. (1) serves as a minimal effective description that captures the essential energetic mechanism governing skyrmion binding, rather than as a complete microscopic Hamiltonian^34,52,53^.

In this study, we numerically investigate the interaction between two skyrmions embedded in a uniformly magnetized background near $B \sim B_\textrm{cr2}$ while varying the skyrmion size by tuning the ratio *D*/*J* in the range $0.5 \le D/J \le 2.5$. To obtain the interaction potential $V(\boldsymbol{R})$, as a function of the skyrmion separation $\boldsymbol{R}$, we fix the positions of the two skyrmions by applying a strong local magnetic field along the $-z$ direction at the desired separation. See Methods for the details of numerical simulations and calculation of the interaction potential. We particularly focus on the interaction potential along the *x* direction, $V(R\boldsymbol{e}_x) \equiv V(R)$, where the deepest attractive minimum is found in our setup^30^. To compare the interaction potentials for different values of *D*/*J*, we normalize the skyrmion separation *R* by the characteristic skyrmion size in the skyrmion-lattice phase obtained from the continuum model^5^, $R_\textrm{sk} = \frac{2\pi J a}{\sqrt{3}D}$, which also represents the size of an isolated skyrmion near $B \sim B_\textrm{cr2}$. Using typical lattice constants of materials that host skyrmions around room temperature, the corresponding skyrmion sizes for the range $0.5 \le D/J \le 2.5$ are estimated to be approximately $R_\textrm{sk} \sim 4$–46 nm^54–56^.

**Fig. 1:**
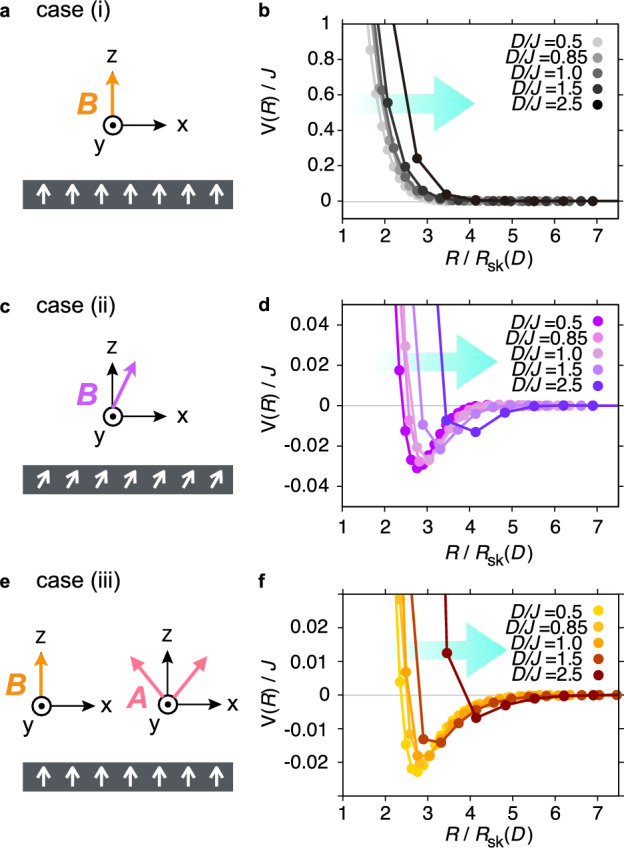
Calculation setups and resulting inter-skyrmion potentials with shape deformation. (**a, c, e**) Schematic images of setups for calculating the inter-skyrmion interactions: (i) under the out-of-plane magnetic field (**a**), (ii) under the tilted magnetic field (**c**), and (iii) under the weak magneto-crystalline anisotropy with the out-of-plane magnetic field (**e**). (**b, d, f**) Inter-skyrmion interaction potentials *V*(*R*) along the *x* direction for different skyrmion sizes $R_\textrm{sk}$ or the Dzyaloshinskii–Moriya (DM) interaction ratios to the exchange interaction *D*/*J* for the setups shown in (**a**, **c**, **e**), respectively. The parameters used are (**b**) $\phi =0^\circ$, $A=0$, and $BJ/D^2=0.75$, (**d**) $\phi =30^\circ$, $A=0$, and $BJ/D^2=0.73$, (**f**) $\phi =0^\circ$, $A/B=0.4$, and $BJ/D^2=0.7$, where $\phi$ is the tilt angle of the magnetic field from the *z*-axis in the *x*-*z* plane, and *A* and *B* are the strengths of the magneto-crystalline anisotropy and the external magnetic field, respectively. As skyrmions shrink, they become stiffer, extending the hard-core-repulsion range (**b**, **d**, and **f**) and shifting the attractive well outward in cases (ii) and (iii) (**d** and **f**), when scaled by the skyrmion radius $R_{\text {sk}}$. In case (ii), a tilt of the background magnetization from ${\boldsymbol{e}}_z$ further enhances the attractive well at larger separations, indicating an additional reinforcing contribution (**d**).

We consider four cases: skyrmions are (i) circular [$\phi =0^\circ$, $A=0$ (Fig. 1a)], (ii) distorted due to a tilted magnetic field [$\phi = 30^\circ$, $A=0$ (Fig. 1c)], (iii) distorted due to a weak magneto-crystalline anisotropy [$\phi =0^\circ$, $A/B=0.4$ (Fig. 1e)], and (iv) a magnetic domain is formed due to a strong magneto-crystalline anisotropy [$\phi =0^\circ$, $A/B=1$ (Fig. 3a)]. The previous study on the inter-skyrmion interaction for sufficiently large skyrmions has shown the following results^30^: in case (i), only hard-core repulsion appears without any attractive well; in cases (ii) and (iii), a small attractive well emerges due to the deformation of the skyrmion shape; and in case (iv), a deep attractive well appears as a magnetic domain is formed between the two skyrmions under a tilted background magnetization at $A/B>0.5$. Below, we separately investigate the skyrmion-size dependence for each mechanism responsible for the appearance of the attractive well.

### Inter-skyrmion potential with shape deformation

 We first investigate the dependence of the inter-skyrmion interaction on the skyrmion size for cases (i)–(iii). Figure 1 shows the inter-skyrmion interaction potential *V*(*R*) as a function of $R/R_{\textrm{sk}}$ for various values of *D*/*J*. In all cases, the qualitative behavior is consistent with that of the larger skyrmions: the attractive well is absent in case (i) (Fig. 1a and b), while it appears in cases (ii) and (iii) due to the deformation of the skyrmion shape (Fig. 1c–f). Quantitatively, the range of the hard-core repulsion increases with increasing *D*/*J*. This is because a skyrmion becomes stiffer against shrinkage as its size approaches the lattice constant. The enhancement of the hard-core repulsion shifts the position of the attractive well outward in cases (ii) and (iii). Moreover, in case (ii), the potential curves do not collapse onto a single curve at large distances ($R/R_{\textrm{sk}}\gtrsim 4$), indicating the presence of an additional mechanism that enhances the attraction at larger distances (Fig. 1d).

**Fig. 2:**
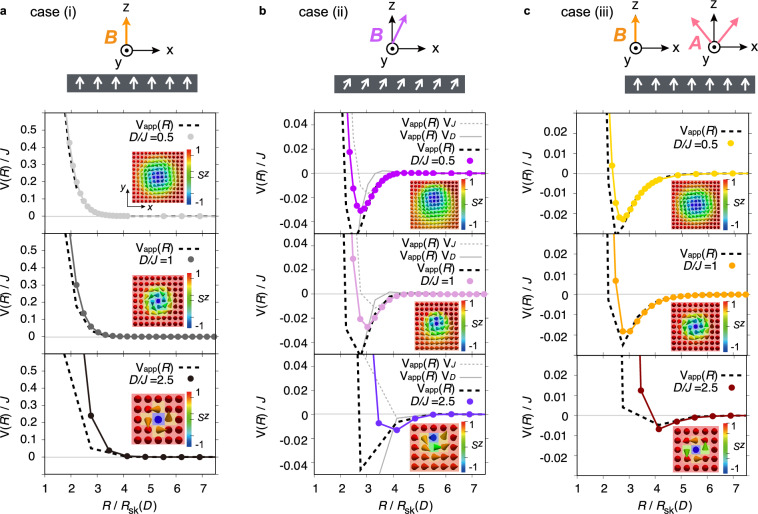
Comparison of simulated and approximated interaction potentials due to shape deformation. (Top panels) Schematic images of setups for calculating the inter-skyrmion interactions. (Lower panels) Comparisons between the numerically obtained interaction potentials, *V*(*R*), and those approximately derived from the single-skyrmion configurations shown in the insets of each panel, $V_\textrm{app}(R)$ (see Methods for details). The panel sizes of the insets are $(1.8R_{\text {sk}})^2$, $(2.5R_{\text {sk}})^2$, and $(3.4R_{\text {sk}})^2$, for $D/J=0.5$, 1, and 2.5, respectively. The data for *V*(*R*) in panels (**a**), (**b**), and (**c**) are the same as those shown in Fig. 1b, d, and f, respectively. In all cases, even for large *D*/*J*, $V_\text {app}(R)$ reproduces *V*(*R*) well at sufficiently large $R/R_\textrm{sk}$. For case (ii) with a tilted background magnetization, $V_\textrm{app}(R)$ consists of two contributions, $V_J(R)$ and $V_D(R)$, which are shown separately in panel (**b**). The DM contribution $V_D(R)$ increases with increasing *D*/*J*, leading to the enhancement of the attractive potential at larger distances well observed in Fig. 1d.

To understand the behavior of *V*(*R*), we consider an approximate expression of the potential at large distances, $V_{\textrm{app}}(R)$, derived from the continuum model. This approximation is obtained by constructing a two-skyrmion state from the magnetization profile of an isolated skyrmion^30^. For the Hamiltonian (1), $V_{\textrm{app}}(R)$ consists of two contributions originating from the exchange interaction, $V_J(R)$, and the DM interaction, $V_D(R)$, which are proportional to *J* and *D*, respectively. The outline of the derivation and the explicit expressions are provided in the Methods section. Figure 2a–c compare *V*(*R*) with $V_{\textrm{app}}(R)$ for cases (i)–(iii), respectively. Here, $V_{\textrm{app}}(R)$ is evaluated using numerically obtained single-skyrmion configurations, which shrink while retaining their overall spatial structure (insets in each panel of Fig. 2). Although $V_{\textrm{app}}(R)$ is based on the continuum approximation, *V*(*R*) and $V_{\textrm{app}}(R)$ show good agreement even for large *D*/*J*, as long as *R* is sufficiently large. This is because the spin texture varies smoothly in regions sufficiently far from the skyrmion center, thereby validating the continuum approximation for $V_{\textrm{app}}(R)$ even for atomic-scale skyrmions. In particular, even in the bottom panels of Fig. 2, corresponding to the largest *D*/*J*, $V_{\textrm{app}}(R)$ reproduces *V*(*R*) well for $R/R_{\textrm{sk}} \gtrsim 4.7$. On the other hand, $V_{\textrm{app}}(R)$ does not necessarily reproduce the numerical results when two skyrmions approach and the magnetization is strongly deformed (i.e., $R/R_{\textrm{sk}} \lesssim 3$ in Fig. 2): $V_{\textrm{app}}(R)$ is based on an approximation valid only when the magnetization does not deviate significantly from the background (see Methods for details). Here, we note that $V_D(R)$ becomes nonzero only when the background magnetization is tilted from the normal direction, $\boldsymbol{e}_z$, which corresponds to the case (ii); hence, the curves for $V_J(R)$ and $V_D(R)$ appear only for case (ii) in Fig. 2b. The contribution of $V_D$, arising from skyrmion deformation, becomes prominent for larger *D*/*J*, i.e., for smaller skyrmion sizes, since $V_D$ scales with *D*. This factor, in addition to the enhancement of the hard-core potential, explains the enhancement of the attractive potential at larger distances observed in Fig. 1d.

### Inter-skyrmion potential with domain formation

**Fig. 3:**
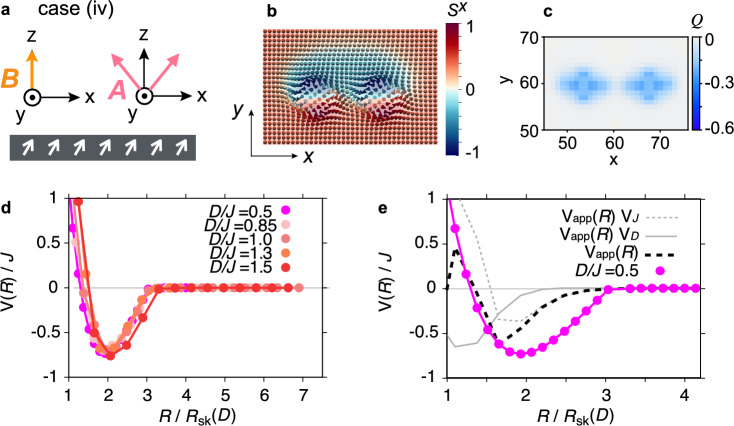
Large attractive wells due to domain formation between skyrmion pairs. (**a**) Schematic of the setup: under an out-of-plane magnetic field, $\phi =0^\circ$, $BJ/D^2=0.7$, and a strong magneto-crystalline anisotropy, $A/B=1.0$, the background magnetization tilts along the *x* direction. (**b**) Stable skyrmion-pair configuration at $D/J = 0.5$, where the color represents the $S^x$ component, and the panel size is $5.3R_\textrm{sk} \times 3.5R_\textrm{sk}$. A magnetic domain with $S^x < 0$ (blue-colored region) emerges, embedded in the background magnetization with $S^x > 0$ (red-colored region) and surrounded by the domain wall (white curve), tightly binding the two skyrmions. (**c**) Spatial distribution of the topological charge density $Q_{\boldsymbol{r}}$, corresponding to the spin configuration in panel (**b**). The integrated topological charge in the displayed region is $-2$, confirming the presence of two skyrmions. (**d**) Inter-skyrmion interaction potential *V*(*R*) for various values of *D*/*J*. Owing to the emergence of a magnetic domain between the skyrmions, the attractive potential becomes very deep, with its depth reaching the order of *J*. Both the depth and the position of the potential well show almost no dependence on *D*/*J*, indicating that the potential is primarily determined by the ratio between the domain area and the domain-wall length. Large *D*/*J* merely changes the potential depth and distance $R/R_{\textrm{sk}}$ which gives the potential minimum. Enhancement of *D*/*J* does not affect the total energy inside the magnetic domain. (**e**) Comparison between $V_\text {app}(R)$ and *V*(*R*) at $D/J=0.5$. Due to the significant deformation of individual skyrmion shapes caused by the domain formation, *V*(*R*) agrees with $V_\textrm{app}(R)$ only when $R/R_\textrm{sk}$ is large enough that the domain does not form.

 Once *A*/*B* exceeds the threshold of 0.5 at $\phi =0^\circ$, the background magnetization gradually tilt toward $\pm \boldsymbol{e}_x$, aligning with one of the two degenerate preferred directions, $\boldsymbol{t}_+$ and $\boldsymbol{t}_-$, determined by the magneto-crystalline anisotropy. We here focus on the background magnetization along ${\hat{\boldsymbol{t}}}_+$ without loss of generality. In the stable configuration, a strong attraction arises between the two skyrmions due to the formation of a magnetic domain polarized along ${\hat{\boldsymbol{t}}}_-$ between them, embedded in the background magnetization oriented along ${\hat{\boldsymbol{t}}}_+$. Figure 3, corresponding to the case (iv), shows that this scenario persists even for smaller skyrmions. In the setup shown in Fig. 3a, the bound state of two skyrmions at $D/J = 0.5$ and $A/B=1.0$ is shown in Fig. 3b, where a blue domain region appears within the red background. The panel illustrates the magnetization structure in the vicinity of the domain wall. This bound state is a stable configuration under the Hamiltonian in Eq. (1). The corresponding topological charge density, $Q_{\boldsymbol{r}}\equiv \frac{1}{4\pi }{\boldsymbol{S}}_{\boldsymbol{r}}\cdot (\partial _x {\boldsymbol{S}}_{\boldsymbol{r}}\times \partial _y {\boldsymbol{S}}_{\boldsymbol{r}})$, displayed in Fig. 3c, yields an integrated value of approximately $-2$, confirming that the configuration consists of two bound skyrmions each carrying a charge of $-1$. A similar domain-mediated configuration is observed for larger *D*/*J*. Figure 3d plots the potential *V*(*R*) as a function of $R/R_\textrm{sk}$ for various values of *D*/*J*. Interestingly, both the depth and position of the attractive well in *V*(*R*) remain nearly unchanged across different *D*/*J*. This robustness indicates that the well depth is governed by the ratio between the domain-wall length and the domain area, which remains roughly constant as the overall size decreases. Figure 3e compares *V*(*R*) with the approximate potential $V_{\textrm{app}}(R)$; once the domain is formed, the strong deformation of individual skyrmions makes $V_{\textrm{app}}(R)$ deviate significantly from the numerical result *V*(*R*), except at sufficiently large distances.

### Effects of the lattice potential on skyrmion-pair dynamics

 In the numerical calculations up to this point, the centers of the skyrmions (where $S^z=-1$) are fixed at the lattice sites. When this constraint is removed and a single-skyrmion state is allowed to evolve in time *t*, the skyrmion center shifts and relaxes to a lower-energy configuration. This indicates that the skyrmion energy depends on its relative position with respect to the underlying lattice sites, meaning that the skyrmion experiences a periodic potential, referred to as the lattice potential, arising from the lattice structure.

For each of the cases (i)–(iv), we estimate the magnitude of the lattice potential, $\Delta E_{\textrm{lattice}}$, by evaluating the energy difference between the single-skyrmion states with and without fixing the skyrmion center; this energy difference is defined as $\Delta E_{\textrm{lattice}}$. In cases (i) and (ii), regardless of the value of *D*/*J*, $\Delta E_{\textrm{lattice}}$ is found to be below $10^{-4}J$ and on the order of 0.01*J*, respectively. In cases (iii) and (iv), under magneto-crystalline anisotropy of $A/B=0.4$ and $A/B=1$, a clear dependence on *D*/*J* is observed, as summarized in Table 1. In both cases, the lattice potential increases monotonically with *D*/*J*.

**Table 1:** Estimated magnitude of the lattice potential $\Delta E_{\textrm{lattice}}$ under magneto-crystalline anisotropy.

*D*/*J*	$\Delta E_{\textrm{lattice}}$, *A*/*B*=0.4	$\Delta E_{\textrm{lattice}}$, *A*/*B*=1
0.85	0.0013*J*	0.17*J*
1.0	0.03*J*	0.39*J*
1.5	0.82*J*	1.8*J*

We next consider the skyrmion dynamics under such a strong lattice potential, focusing on case (iv). To this end, we numerically investigate the relaxation dynamics of a skyrmion pair toward its stable separation, starting from an initial distance larger than $R_\textrm{min}$, the separation corresponding to the potential minimum. In the absence of the lattice potential, the two skyrmions would relax to a stationary state at the separation $R_\textrm{min}$. However, the presence of the lattice potential is expected to hinder their motion. In particular, while the inter-skyrmion interaction potential shows little dependence on *D*/*J* (see Fig. 3d), the lattice potential becomes stronger with increasing *D*/*J*. Consequently, smaller skyrmions (i.e., those with larger *D*/*J*) are likely to be pinned by the lattice potential before reaching $R_\textrm{min}$.

**Fig. 4:**
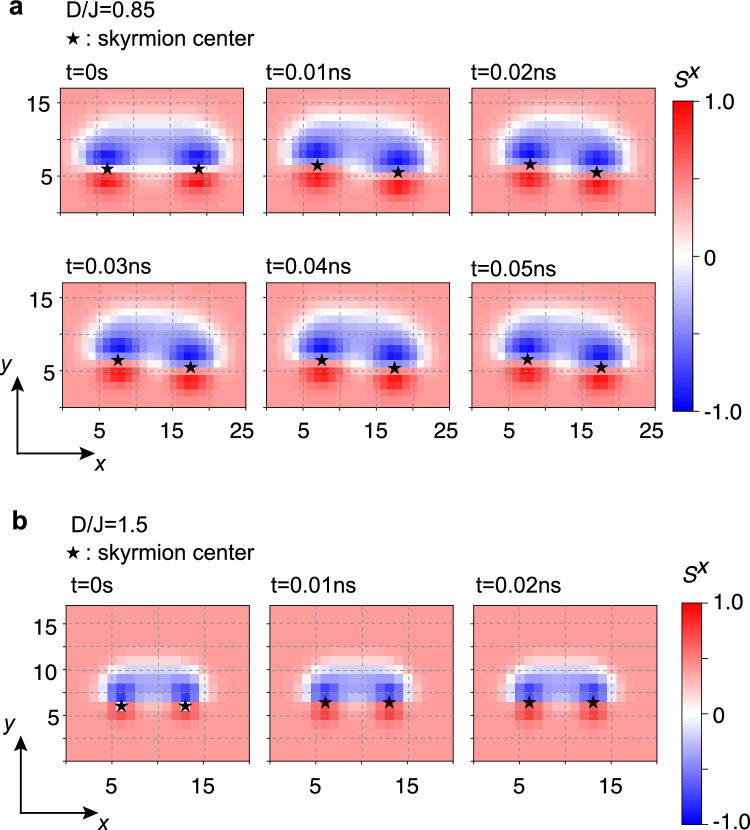
Lattice-potential pinning of skyrmion pairs. Snapshots of the relaxation process of skyrmion pairs in case (iv), under strong magneto-crystalline anisotropy of $A/B = 1$. At time $t=0$ s, the initial separation is set to $R\sim 3R_\textrm{sk}$, exceeding the potential minimum $R_\textrm{min}\sim 2R_\textrm{sk}$. Black stars denote the center sites of each skyrmion at each time step. (**a**) For $D/J=0.85$, the pair moves toward the minimum: the centers shift from (6*a*, 6*a*) and (19*a*, 6*a*) to (7.5*a*, 6.5*a*) and (17.5*a*, 5.5*a*). The final separation $\boldsymbol{R}=10a\boldsymbol{e}_x+a\boldsymbol{e}_y$, however, remains larger than the minimum $\boldsymbol{R}_\textrm{min}=9a\boldsymbol{e}_x$. This is because the attractive force $-\boldsymbol{\nabla }V(\boldsymbol{R})$ weakens near the minimum, allowing lattice pinning. (**b**) For $D/J = 1.5$, influence of the lattice potential becomes stronger (see Table 1). The lattice pinning prevents the pair from closing the gap despite strong attraction: the centers move from (6*a*, 6*a*) and (13*a*, 6*a*) to (6*a*, 6.5*a*) and (13*a*, 6.5*a*), and the final separation $\boldsymbol{R}=7a{\boldsymbol{e}}_x$ remains larger than $\boldsymbol{R}_\textrm{min}=5a{\boldsymbol{e}}_x$. The pair shifts by half a lattice constant along the *y* direction, with each skyrmion settling at a lattice-potential minimum.

We indeed observe such pinning by the lattice potential in the numerical simulations. Figure 4 shows snapshots of the skyrmion pair during the relaxation dynamics for $D/J = 0.85$ (Fig. 4a) and 1.5 (Fig. 4b) under a strong magneto-crystalline anisotropy of $A/B = 1$. In both cases, the initial separation is set to $R \sim 3R_\textrm{sk}$, which is larger than $R_\textrm{min} \sim 2R_\textrm{sk}$. As shown in Fig. 4a, for $D/J = 0.85$, the two skyrmions move toward the potential minimum, with their centers shifting from (6*a*, 6*a*) and (19*a*, 6*a*) to (7.5*a*, 6.5*a*) and (17.5*a*, 5.5*a*), respectively. Here, the skyrmion center, denoted by a black star at each time step, is defined as the point where $S^z$ takes its minimum value. When multiple sites have $S^z$ values that differ by less than 0.01 (as in the present final state), the center of the skyrmion is considered to be at the average position of these sites. The final skyrmion separation of $\boldsymbol{R}=10a\boldsymbol{e}_x + a\boldsymbol{e}_y$ does not reach the exact minimum of the interaction potential at $\boldsymbol{R}_\textrm{min}=9a\boldsymbol{e}_x$. This behavior occurs because the attractive force, $-\boldsymbol{\nabla } V(\boldsymbol{R})$, becomes weaker near the potential minimum, allowing the skyrmions to be pinned by the lattice potential. As shown in Fig. 4b, for $D/J=1.5$, the pinning effect becomes even more pronounced: their centers shift from (6*a*, 6*a*) and (13*a*, 6*a*) to (6*a*, 6.5*a*) and (13*a*, 6.5*a*), respectively. The final separation, $\boldsymbol{R} = 7a\boldsymbol{e}_x$, remains larger than the potential minimum $\boldsymbol{R}_\textrm{min} = 5a\boldsymbol{e}_x$. The skyrmion pair shifts by half a lattice constant along the *y* direction so that each skyrmion reaches the minimum of the lattice potential, while maintaining their mutual separation. The shift originates from the potential shape, reflecting the symmetry of the model Eq. (1); the potential minimum does not necessarily coincide with a lattice site, but can instead correspond to a high-symmetry position shifted along the *x* and/or *y* directions. While Fig. 4 illustrates cases where the pinning effect is pronounced, we find that skyrmion pinning also occurs under a weaker lattice potential. At large *R*, where the driving force originating from the skyrmion-skyrmion interaction becomes sufficiently weak, the skyrmions are trapped by the lattice potential and remain immobile even in cases (i)–(iii). We also comment that the skyrmions at $D/J=2.5$, shown in Figs. 1 and 2, disappear once the constraint fixing the skyrmion position is removed. This indicates that the attractive potentials discussed in the previous sections should be interpreted as those for skyrmions stabilized under pinning conditions.

## Discussion

In conclusion, we have examined the interactions between atomic-scale skyrmions, embedded in a uniformly magnetized background, focusing on how the interaction mechanisms observed in large skyrmions persist in the small-skyrmion regime. The numerical analyses are performed for four cases: (i) under an out-of-plane magnetic field (Fig. 1a); (ii) with background magnetization tilted by an in-plane field (Fig. 1c); (iii) under weak magneto-crystalline anisotropy (Fig. 1e); and (iv) with background magnetization tilted by strong magneto-crystalline anisotropy (Fig. 3a). In all cases, the large-separation potential matches the approximate expression of the potential $V_\textrm{app}(R)$. In cases (i)–(iii), when scaled by the skyrmion size $R_\textrm{sk}$, the small skyrmions become stiffer and the hard-core repulsion extends. In cases (ii) and (iii), the shape deformation still induces attractive potential wells even for small skyrmions, consistent with the prediction from the continuum model. Especially in case (ii), the tilted background magnetization strengthens the attractive well at larger $R/R_\textrm{sk}$, due to an additional contribution to $V_\textrm{app}(R)$. Under strong magneto-crystalline anisotropy, corresponding to case (iv), a strong attraction, comparable to the exchange interaction, emerges even between the small skyrmions. Both the depth and position of the attractive well remain nearly independent of $R_\textrm{sk}$, indicating that the potential shape is determined by the ratio between domain-wall length and domain area. These features are expected to persist as long as a magnetic domain forms between two skyrmions, and are not specific to the particular cubic anisotropy or geometry considered in our model. The influence of the lattice potential becomes more pronounced as the skyrmion size decreases (Table 1), leading to lattice pinning that hinders skyrmion motion. Once the lattice potential dominates, skyrmion pairs cannot reduce their separation despite strong attraction (Fig. 4).

These results may advance the use of small skyrmions as information carriers in magnetic devices and show that attractive wells may control atomic-scale skyrmion motion. In cases of strong magnetic anisotropy where magnetic domains form, the binding energy of skyrmion pairs can reach the order of *J*. For materials with $J \sim 25\,\textrm{meV}$, such bound skyrmions are expected to remain stable against thermal fluctuations even at room temperature. Furthermore, the strength of the attractive interaction between bound skyrmion pairs can be continuously and reversibly tuned by an external magnetic field^30^. Exploiting this tunability, skyrmion states with different interaction strengths may be interpreted as weighted synaptic elements. In addition, the lattice potential may make skyrmion clustering and positional pinning more robust, which is expected to enhance the stability of certain types of device operations. Also, the approximate potential $V_{\textrm{app}}(R)$ remains a useful guide to deformation-induced interactions. Several aspects are left for the future work: the effects of thermal fluctuations and impurities on interactions and relaxation; the incorporation of the actual two-dimensional lattice structure of the (011) plane beyond the square-lattice approximation used in this study; and direct numerical studies of systems with bilinear, biquadratic, and frustrated exchange interactions, which are considered the primary origin of atomic-scale skyrmions^34,36–41,52,53^.

## Methods

### Computing skyrmion stationary states and their relaxation dynamics

 Starting from an initial configuration with one or two skyrmions embedded in a uniformly magnetized background, we evolve the system according to the Landau–Lifshitz–Gilbert equation at 0 K,

2 $$\begin{aligned} \frac{d{\boldsymbol{S}}_{\boldsymbol{r}}}{dt} = -{\boldsymbol{S}}_{\boldsymbol{r}}\times {\boldsymbol{B}}_{\textrm{eff}} + \alpha {\boldsymbol{S}}_{\boldsymbol{r}}\times \frac{d{\boldsymbol{S}}_{\boldsymbol{r}}}{dt}, \end{aligned}$$

where $\boldsymbol{B}_\textrm{eff}=-\delta H/\delta {\boldsymbol{S}}_{\boldsymbol{r}}$ is the effective magnetic field and $\alpha =0.1$ is the damping constant. To obtain skyrmion stationary states, we relax the state until the energy change drops below $10^{-5}J$. Numerical simulations are carried out using a customized version of the open-source software JAMS^57^.

### Numerical calculation of the skyrmion-skyrmion interaction

 To calculate the interactions between two skyrmions, embedded in a uniform background magnetization along $\hat{\boldsymbol{t}}$, we compare energies as follows. We evaluate the energy of a single skyrmion, $E_\text {1sk}$, to that of two skyrmions separated by a relative distance ${\boldsymbol{R}}$, $E_\text {2sk}({\boldsymbol{R}})$, with respect to the energy of the background uniform configuration ${\boldsymbol{S}}_{\boldsymbol{r}}={\hat{\boldsymbol{t}}}$, $E_\text {ferro}$. The skyrmion-skyrmion interaction reads

3 $$\begin{aligned} V({\boldsymbol{R}})=E_\text {2sk}({\boldsymbol{R}})-2E_\text {1sk}+E_\text {ferro}. \end{aligned}$$

### Approximate interaction potential at large distances

 We briefly summarize the results obtained in the continuum limit^30^. Consider a stable magnetization profile $\boldsymbol{n}(\boldsymbol{r})$ in a thin magnetic film. Here, $\boldsymbol{n}(\boldsymbol{r})$ is taken to be a continuous function of $\boldsymbol{r}=(x,y)$, which agrees at each lattice site with the normalized magnetization vector $\boldsymbol{S}_{\boldsymbol{r}}$ defined there. Suppose that the film is uniformly magnetized along the direction $\hat{\boldsymbol{t}}$, with a pair of skyrmions embedded as excitations. For sufficiently large separations, a two-skyrmion configuration can be constructed from the stationary magnetization profile of a single skyrmion, $\boldsymbol{n}_\textrm{1sk}(\boldsymbol{r})$, whose origin is set to $\boldsymbol{n}_\textrm{1sk}(\boldsymbol{0}) = -\hat{\boldsymbol{t}}$. From this construction, the inter-skyrmion interaction energy can be analytically obtained in terms of $\boldsymbol{n}_\text {1sk}(\boldsymbol{r})$ as a function of the separation $\boldsymbol{R}$. The interaction originates from the gradient-dependent terms in the energy functional. Under the Hamiltonian (1), the only gradient-dependent terms in the energy functional are the exchange and DM interactions, whose energy densities are given respectively by $Ja^2/2|\nabla \boldsymbol{n}|^2$ and $Da\boldsymbol{n}\cdot (\boldsymbol{\nabla }\times \boldsymbol{n})$. In this case, the interaction potential between two skyrmions aligned along the *x* direction is given by

4 $$\begin{aligned}&V_\text {app}(R{\boldsymbol{e}}_x)=V_J(R{\boldsymbol{e}}_x)+V_D(R{\boldsymbol{e}}_x),\end{aligned}$$

5 $$\begin{aligned}&V_J(R{\boldsymbol{e}}_x)=2J\int _{-\infty }^\infty \left[ \partial _x m_x\left( \frac{R}{2},y\right) \right] m_x\left( -\frac{R}{2},y\right) dy+2J\int _{-\infty }^\infty \left[ \partial _x m_y\left( \frac{R}{2},y\right) \right] m_y\left( -\frac{R}{2},y\right) dy,\end{aligned}$$

6 $$\begin{aligned}&V_D(R{\boldsymbol{e}}_x)=\frac{2D}{a} (\hat{\boldsymbol{t}}\cdot \boldsymbol{e}_x)\int _{-\infty }^\infty dy\left[ m_x\left( -\frac{R}{2},y\right) m_y\left( \frac{R}{2},y\right) -m_x\left( \frac{R}{2},y\right) m_y\left( -\frac{R}{2},y\right) \right] , \end{aligned}$$

where $m_x$ and $m_y$ are the components of $\boldsymbol{n}_\text {1sk}(\boldsymbol{r})$ projected onto the perpendicular plane to ${\hat{\boldsymbol{t}}}$, such that $m_x=n_x\cos \theta -n_z\sin \theta$ and $m_y=n_y$, where $\theta \equiv \arctan [({\boldsymbol{e}}_x\cdot {\hat{\boldsymbol{t}}})/({\boldsymbol{e}}_z\cdot {\hat{\boldsymbol{t}}})]$ takes the principal value. Between the two components in Eq. (4), $V_J$ is nonzero in all cases (i)–(iv). In contrast, $V_D$ becomes nonzero only when the background magnetization ${\hat{\boldsymbol{t}}}$ is tilted from the normal direction ${\boldsymbol{e}}_z$, i.e., when $\hat{\boldsymbol{t}}\cdot \boldsymbol{e}_x\ne 0$, which occurs only in case (ii). In the continuum model, both term give rise to an attractive well when skyrmions are distorted, for example, by a tilted external magnetic field or by magneto-crystalline anisotropy^30^.

## Data Availability

The data that support the findings of this study are available from the corresponding author upon reasonable request.

## Code availability

The code is available from the corresponding author upon reasonable request. Most parts of the code are already publicly available on GitHub^57^.
